# Involuntary Entry Into Consciousness From the Activation of Sets: Object Counting and Color Naming

**DOI:** 10.3389/fpsyg.2018.01017

**Published:** 2018-06-21

**Authors:** Sabrina Bhangal, Christina Merrick, Hyein Cho, Ezequiel Morsella

**Affiliations:** ^1^Department of Psychology, University of Missouri, Columbia, MO, United States; ^2^Department of Psychology, University of California, Berkeley, Berkeley, CA, United States; ^3^Department of Psychology, The Graduate Center, City University of New York, New York, NY, United States; ^4^Department of Psychology, San Francisco State University, San Francisco, CA, United States; ^5^Department of Neurology, University of California, San Francisco, San Francisco, CA, United States

**Keywords:** consciousness, set-based entry, involuntary processing, reflexive imagery task, stimulus control

## Abstract

High-level cognitions can enter consciousness through the activation of certain action sets and the presentation of external stimuli (“set-based entry,” for short). Set-based entry arises in a manner that is involuntary and systematic. In the Reflexive Imagery Task, for example, subjects are presented with visual objects and instructed to not think of the names of the objects. Involuntary subvocalizations arise on roughly 80% of the trials. We examined whether or not set-based entry can also occur in the case of involuntary counting. Subjects in Experiment 1A were instructed to not count the number of objects presented in an array. Involuntary counting arose on a high proportion of the trials (a mean proportion of ∼0.90) for stimulus arrays having 2–5 objects, and such counting arose less frequently across trials when the array consisted of 6–10 objects (a mean proportion of ∼0.21). The data from Experiment 1B revealed that, when people choose to perform Set *X*, they also experience thoughts about an unselected Set (Set *Y*). Subjects were trained on one set (e.g., to “color name”) and then, when presented with stimuli, were given the choice to perform the trained set or a novel set. Consistent with theories proposing that the conscious contents represent several potential action plans, subjects were equally likely to experience set-related imagery or set-unrelated imagery. Our findings regarding set-based entry are relevant to many subfields of psychology and neuroscience (e.g., the study of high-level mental processes, attention, imagery, and action control).

## Introduction

Understanding the mechanisms underlying the phenomenon of “entry into consciousness" (“entry," for short; Di Lollo et al., 2000; Mathewson et al., 2009) remains one of the most daunting puzzles in science (Crick and Koch, 2003). Entry is influenced by various processes, including those that are voluntary, such as choosing to think about certain things, or attention-based (see review in Most et al., 2005). Recent research has begun to illuminate the nature of the various kinds of mechanisms underlying entry that is involuntary. This form of entry can arise from the salience, motion, novelty, or incentive/emotional quality of the stimulus (Gazzaley and D’Esposito, 2007; Goodhew, 2017). Involuntary entry can be of percepts, urges (Morsella et al., 2016), or even high-level cognitions.

Regarding high-level cognitions (e.g., mental imagery and subvocalizations), the involuntary entry of these cognitions can arise as a consequence of the activation of *action sets*, the topic of the present research project. An action set would be “when perceiving *X*, then do *Y*” (Ach 1905/1951), for example, “when I see a mailbox, I must deposit the letter that I am carrying.” Regarding action sets, Ach (1905/1951) speaks of the example in which, after activating the action set to “add things” and being presented with the numbers two and six, there is the involuntary entry of the *conscious content* “eight.”^1^ In this way, entry of high-level conscious contents can arise from the activation of action sets (“set-based entry,” for short). Theorists (e.g., Freud, 1938; Helmholtz, 1856/1925; James, 1890; Miller, 1959; Wegner, 1989) have proposed that, during such involuntary entry, one is conscious only of the product (e.g., the phonological form “eight”) of sophisticated processes, a view that has recurred in the history of psychology (e.g., Lashley, 1956; Miller, 1962).

The Reflexive Imagery Task (RIT; Allen et al., 2013b) was developed to investigate experimentally involuntary set-based entry. We review this paradigm in the next section.

## Reflexive Imagery Task

The RIT (see review in Bhangal et al., 2016b) is based on a rich research tradition, stemming from the experimental approaches of Ach (1905/1951), Stroop (1935), Uznadze (1966), Wegner (1989), and Gollwitzer (1999). The paradigm was developed to investigate experimentally the involuntary entry of high-level conscious contents. In the initial, most basic version of the task (Allen et al., 2013b), subjects are instructed to not subvocalize (i.e., say in their head but not aloud) the names of objects (e.g., line drawings from Snodgrass and Vanderwart, 1980). In Allen et al. (2013b), subjects were presented before each trial with the instruction, “Don’t Think of the Name of the Object” before an object was presented for 4 s, during which time subjects indicated by button press if they happened to subvocalize the name of the object. On the majority of the trials (86% in Allen et al., 2013b; 87% in Cho et al., 2014; and 73% in Merrick et al., 2015), subjects fail to suppress such subvocalizations. To illustrate the basic version of the RIT effect, momentarily, we will present to you, the reader, an object enclosed within parentheses. Your task is to *not* subvocalize (i.e., “say in one’s head”) the name of the object. Here is the stimulus (▴). When presented with these instructions (which induce a certain action set) and then presented by this stimulus, most people cannot suppress the conscious experience of the phonological form of the word “triangle.”^2^

There are more complex versions of the task. In one study, RIT effects arose even though the involuntary effect involved a word-manipulation task similar to the childhood game of Pig Latin (e.g., “CAR” becomes “AR-CAY”). In this variant of the RIT (Cho et al., 2016), subjects were instructed to not transform stimulus words according to the rule. Involuntary transformations still arose on more than 40% of the trials. This set-based effect is noteworthy because the involuntary transformation of the word stimulus requires symbol manipulation, a complex operation which is known to be associated with frontal cortex (Miller and Cummings, 2007).

## Validity of Subjects’ Self-Reports

Questions remain concerning the validity of the RIT effect. For instance, one criticism is that the paradigm relies on the technique of self-report. Self-reports can be inaccurate as a result of (a) inaccurate memories of fleeting conscious contents that lead to incorrect self-reports (Block, 2007), or (b) subjects basing their reports on a strategy of how to comport oneself during an experiment (see discussion in Morsella et al., 2009a). Evidence from neuroimaging studies suggests that subjects are not confabulating about the occurrence of these mental events. In these studies, subjects must report about the occurrence of involuntary conscious contents (Wyland et al., 2003; Mason et al., 2007; Mitchell et al., 2007; McVay and Kane, 2010; Pasley et al., 2012). Strong behavioral evidence for subjects’ self-reports stems from Cushing et al. (2017). In this experiment, subjects indicated by button press the basic RIT effect but, in addition, they had to press another button if the involuntary subvocalization rhymed with a word held in mind. Accurate performance (>80% mean accuracy across trials) on this rhyming task provided evidence that subjects do experience involuntary subvocalizations of the name of the object, for detecting a rhyme requires the retrieval of either the whole object name or, at the least, the coda of the word of the object name.

## Evidence That the Effect Resembles a Reflex

In one version of the RIT, subjects reported on the majority of trials that the involuntary subvocalization felt “immediate” (Bhangal et al., 2015). This is consistent with empirical evidence and theory [including Wegner’s (1994) model of *ironic processing*] suggesting that, for subjects, the effect should “just happen.” (An ironic process occurs when one is more likely to think about a given thing when attempting to not think about that thing.) The effect does not seem to be an artifact of high-level strategic processes. First, on many trials, the effect arises too quickly to be caused by strategic processing (Allen et al., 2013b; Cho et al., 2014). Second, the nature of the subvocalizations is influenced systematically by stimulus dimensions such as word frequency (Bhangal et al., 2015). Such an artifact of experimental demand would require for subjects to know the ways in which word frequency should influence latencies in an object-naming experiment. Third, the effect habituates (i.e., is less likely to arise) after repeated presentation of the same stimulus object, which suggests that the RIT effect is activated in a reflex-like manner (Bhangal et al., 2016a). Last, the RIT effect still arises under conditions of cognitive load, in which it is difficult for subjects to implement strategic processing (Cho et al., 2014).

## The Rit Effect and Ironic Processing

Wegner (1994) proposes that ironic effects^3^ arise from an interaction between two distinct processes [see discussion of relationship between Wegner’s (1994) model and the RIT in Bhangal et al., 2016b]. One process is an *operating* process, which is associated with the conscious intention to maintain a particular mental state. This process actively scans mental contents (e.g., thoughts, sensations) that can help maintain the desired mental state (e.g., to be calm). This process tends to be effortful, capacity-limited, and consciously mediated (Wegner, 1994). The other mechanism is an “ironic” *monitoring* process that automatically scans activated mental contents to detect contents signaling the failure to establish the desired mental state. When the monitor detects contents that signify failed control of the operating mechanism, it increases the likelihood that the particular content will enter consciousness, so that the operating mechanism can then process the content and change its own operations accordingly. The ironic monitor mechanism is usually unconscious, autonomous, and requires little mental effort. Harmony between the two kinds of processes fails when the goal in mental control is to *not* activate a particular mental content (e.g., content *X*), because (a) the operating process can bring only goal-related contents into consciousness and cannot actively exclude contents, and (b) the ironic monitor will reflexively bring into consciousness mental contents (e.g., content *X*) that are incongruent with the goal. Hence, there will be the automatic activation of content *X* in consciousness (for reviews of ironic processing and thought suppression, see Rassin, 2005; Wegner, 1989).

Germane to Wegner’s views of the monitor bringing undesired contents into consciousness, in the basic RIT involving involuntary subvocalizations in response to the presentation of visual stimuli, the following might be transpiring. The subject, in order to monitor whether his or her performance was successful on a given trial, might employ the practice of imagining what the undesired mental operation would be (e.g., the name “triangle”). Conceptually, this imagination of the undesired outcome (a simulation of sorts) could be construed as qualitatively distinct from the actual execution of the (undesired) mental operation. For the subject, the activation of such a mental representation of a simulacrum, which simulates what the undesired mental operation would be, might be indistinguishable from the representation resulting from the actual undesired mental operation, leading to the illusion that the undesired mental operation was executed.

This hypothesis, however, appears to be incapable of accounting for RIT effects requiring mental operations that are more complex in nature. The simulacrum for these variants of the RIT would require the actual execution of the undesired mental operation. For example, in Cho et al. (2016), RIT effects arose when the involuntary effect required symbol manipulations similar to those of the childhood game of Pig Latin (e.g., “CAR” becomes “AR-CAY”). Any simulacrum of the undesired end-state (“AR-CAY”) would require the execution of the undesired, syntactic mental operation. Similarly, in Merrick et al. (2015), subjects were presented with a single, focal object (DOOR) and instructed to (a) not subvocalize the name of the visual object, and (b) not subvocalize the number of letters in the object name (e.g., “four”). On a considerable proportion of the trials [*M* = 0.30 (*SE* = 0.04)], subjects reported experiencing both kinds of imagery. Importantly, in that experiment, any simulacrum of the undesired thoughts required the execution of the two unintentional cognitive processes: object naming versus letter counting.^4^

## Extension of the Rit Into the Realm of Involuntary Counting

Previous research has conveyed that the automatic counting of visual objects (often referred to as “subitizing”; Kaufman et al., 1949) is more likely to occur when items range from 1 to 5 (Trick and Pylyshyn, 1994). In the current study, we wanted to investigate whether or not such automatic effects would arise even when subjects are instructed explicitly to “not count the number of objects presented.” To our knowledge, no study to date has used the RIT paradigm to examine this automatic phenomenon involving counting and the activation of undesired action sets. The number of visual objects in the stimulus arrays presented to subjects was either within the range for automatic counting (i.e., 2–5 objects; Trick and Pylyshyn, 1994) or outside this range (i.e., 6–10 objects). We predicted that more RIT effects (i.e., involuntary counting) would occur for the former than for the latter.

As in Bhangal et al. (2015), we took the opportunity to measure on a trial-by-trial basis whether subjects perceived the effect of involuntary counting to be “immediate.” Previous RIT studies have shown that the involuntary entry into consciousness of contents is often perceived by the subject to be immediate (e.g., in 75% of trials in Bhangal et al., 2015). Thus, we predicted that RIT effects (i.e., involuntary counting) occurring for arrays in the subitizing range would more often be perceived as immediate compared to RIT effects occurring in the condition in which 6–10 objects are presented.

When creating the stimulus arrays, we took the opportunity to manipulate whether all the visual objects were presented in the same color (color uniformity condition) or not (disuniform condition), as such stimulus properties are known to influence the nature of involuntary counting (Frick, 1987; Puts and de Weert, 1997; Watson and Maylor, 2006). For example, some previous findings (e.g., Watson and Maylor, 2006) suggest that the color of the objects has no influence on the speed of subitizing. However, other experiments have found that (a) objects presented in the same color will lead to slower forms of subitizing (Puts and de Weert, 1997; Trick, 2008), or (b) objects that are presented in different colors will lead to slower forms of subitizing (Frick, 1987).

## Experiment 1A: Involuntary Entry From the Unselected Set to Count Objects

### Method

#### Subjects

San Francisco State University students (*n* = 34, *M*_Age_ = 23.56, *SD*_Age_ = 5.26, and females = 22) participated for course credit. The involvement of human subjects in our project was approved by the Institutional Review Board at San Francisco State University. For this and all subsequent studies, subjects provided written informed consent at the beginning of the experimental session.

#### Stimuli and Apparatus

**FIGURE 1:**
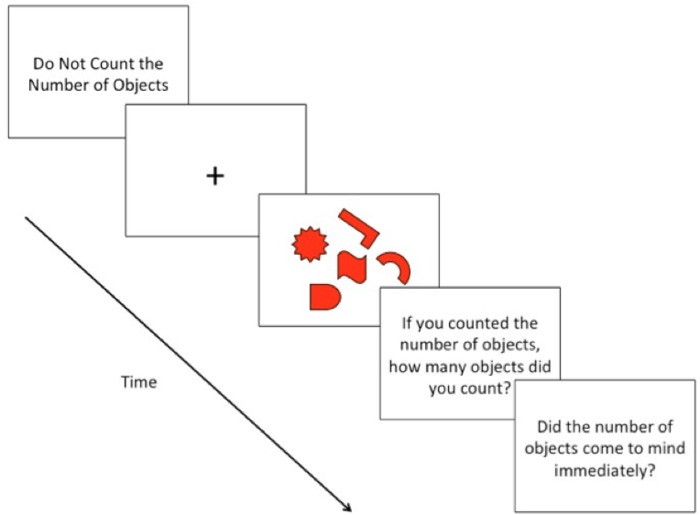
Schematic depiction of a typical trial with an array of objects that are all presented in the same color.

**FIGURE 2:**
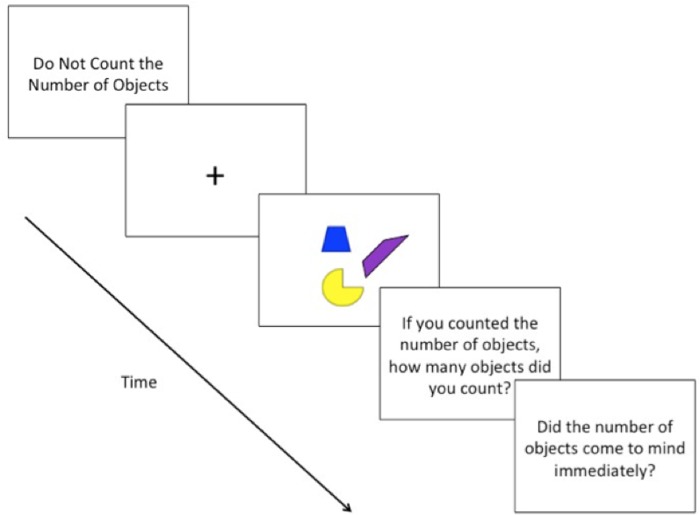
Schematic depiction of a typical trial with an array of objects presented in different colors.

Stimuli consisted of non-readily nameable shapes selected from *Shape* in Microsoft PowerPoint for Mac (2011) version 14.1. The shapes in the study had been used successfully in a previous study (Allen et al., 2013a). The stimulus arrays were randomized and varied on two dimensions: color [black (RGB: 0, 0, 0), blue (0, 0, 255), brown (153, 102, 51), green (0, 255, 0), orange (255, 127, 0), pink (255, 97, 238), purple (127, 0, 127), red (255, 0, 0), white (255, 255, 255), and yellow (255, 255, 0)] and the number of objects presented in the array (2, 3, 4, 5, 6, 7, 8, 9, and 10), yielding 72 unique arrays. Half of the trials consisted of object arrays that were presented in the same color (**Figure 1**), while the other half of the trials consisted of object arrays presented in various types of colors (**Figure 2**). The order of presentation of object arrays was randomized. Stimuli consisted of arrays that were within the subitizing range (2–5 objects) or outside of the subitizing range (6–10 objects). The placement of the objects within each array formed a distinct pattern, which differed from that of all other arrays for each specific subitizing range.

Each object shape was outlined with a solid black border (3/4 pt) to make the objects more perceivable to the subject. The shades and colors of the objects had been used successfully in previous studies (Morsella et al., 2009a) and, according to the funneled debriefing questionnaire (see below), no subject reported that he or she could not perceive the colors of the objects. All objects were presented in an array with a white background, on an Apple iMac computer monitor (50.8 cm) with a viewing distance of approximately 48 cm. Arrays were displayed within a centered visual angle of 11.89° × 16.59° (10 × 14 cm) (see **Appendix** for a complete list of visual angles for each object range). Each object presented in a stimulus array occupied a region of 3.5 cm high and 3.5 cm wide (5.96° × 5.96°). For all experiments, stimuli were presented at the setting of maximum brightness on the Apple iMac computer. Stimulus presentation and data recording were controlled by PsyScope software (Cohen et al., 1993). All questions and instructions were written in black 36-point Helvetica font on a white background.

#### Procedures

Instructions, presented on the computer screen, informed subjects that they would be shown a series of object arrays. Each subject was presented with 72 trials, 32 of which presented arrays having a number of objects within the subitizing range, and 40 of which presented arrays having a number of objects outside of this range. Half (*n* = 16) of the arrays having stimuli within the subitizing range had all of the stimuli presented in a uniform color; the other half did not. Similarly, half (*n* = 20) of the arrays having stimuli outside of the subitizing range presented all the objects in a uniform color; the other half did not. The Subitizing Range condition and Color condition were not blocked but intermixed, with each of the 72 stimuli presented in random order across 72 trials.

Before each trial, the phrase “*Do Not Count the Number of Objects*” was displayed in the center of the screen, serving as a ready prompt; subjects indicated their readiness by pressing the return key. Once subjects indicated their readiness, a fixation-cross (+) appeared in the center of the screen (700 ms), preparing subjects for the presentation of the array of objects (**Figures 1** and **2**). Following the presentation of the fixation-cross, the array of objects appeared for 4 s, based on the stimulus presentation time of Allen et al. (2013b). During the time of the stimulus presentation, subjects could indicate, by pressing the spacebar, if they happened to count the number of objects. It was stressed to subjects to press the spacebar once, and as quickly as possible, only if they happened to count the number of objects that appeared on the screen. Subjects were instructed to keep, at all times, their eyes focused on the center of the screen and to keep their left hand rested on the spacebar. Subjects were also instructed not to respond in any way if they did not happen to count the number of objects. Subjects were informed that the objects would remain on-screen for a fixed amount of time, regardless of whether or not they pressed the spacebar.

After each trial, subjects were presented with two questions: “*If you counted the number of objects, how many objects did you count? If you did not count the number of objects, you may indicate this by pressing ‘0’*” and “*If you counted the number of objects, did you feel that the number of objects came to mind immediately?”* In response to the first question, subjects typed the number of objects they counted using the number pad on the keyboard. In response to the second question, subjects indicated their response by typing *y* for “yes” and *n* for “no.”

Before performing the critical trials, subjects first completed four practice trials in which they were instructed to not count the number of objects. The practice trials resembled the critical trials. The object arrays used during the practice trials remained the same across subjects. The object arrays used in the practice trials were distinct from the object arrays presented during the experimental trials.

Once subjects completed the experiment, they responded to a series of funneled debriefing questions (following the procedures of Bargh and Chartrand, 2000), which included general questions to assess whether subjects (a) were aware of the purpose of the study, (b) had any strategies and/or goals for completing the task, (c) had anything interfere with their performance on the task, and (d) tried their best to follow instructions. These questions were used to determine if the data collected from the subjects were adequate enough to be used for the analyses.

The data from five subjects were excluded from analysis because it was obvious to the experimenter that these subjects were not following instructions (i.e., always reporting the incorrect number of objects on trials in which they reported that they subitized the number of objects, not pressing the spacebar to indicate that they had counted the number of objects in the array even though they reported the number of objects they subitized), and in one case, the experimental session was terminated due to a computer malfunction.

### Results

#### Proportion of Involuntary Counting Across Trials

The mean proportion of trials in which involuntary counting occurred was 0.90 (*SD* = 0.11, *SE* = 0.02) for the condition in which the objects were within the subitizing range (2–5 objects) and were of uniform color. The rate was 0.91 (*SD* = 0.12, *SE* = 0.02) for the condition in which the objects were within the subitizing range and not of uniform color. When the number of objects was outside the subitizing range (i.e., 6–10 objects), the mean rates of involuntary counting decreased: 0.24_Uniform Color_ (*SD* = 0.24, *SE* = 0.04), 0.19_Disuniform Color_ (*SD* = 0.21, *SE* = 0.04).

In a fully within-subjects ANOVA with Range (within versus outside) as one factor and Color (same versus different) as the other factor, there was a significant main effect of Range, *F*(1,33) = 511.49, *p* < 0.0001, in which the rate for involuntary counting was higher when the objects were within the subitizing range than when the objects were outside the subitizing range. There was no main effect of Color *F*(1,33) = 4.18, *p* = 0.05. There was a significant interaction between the two factors, *F*(1,33) = 9.31, *p* < 0.01. This interaction requires replication and further investigation. Of import, this unexpected interaction effect does not qualify our primary conclusions in any substantive way.

Similar effects were found when the analyses excluded the data from trials in which the subject miscounted the objects. When the number of stimuli were within the subitizing range, involuntary counting occurred on an average proportion of 0.97 of the trials (*SD* = 0.07) in the Uniform Color condition and 0.97 (*SD* = 0.05) in the Disuniform Color condition. When the number of stimuli fell outside the subitizing range, involuntary counting occurred on 0.73 (*SD* = 0.30, *SE* = 0.06) of the trials in the Uniform Color condition and 0.86 (*SD* = 0.27, *SE* = 0.05) of the trials in the Disuniform Color condition. In a fully within-subjects ANOVA with Range (within versus outside) as one factor and Color (same versus different) as the other factor, there was a significant main effect of Range, *F*(1,23) = 17.91, *p* < 0.001, in which the rate for involuntary counting was higher when the objects were within the subitizing range than when the objects were outside the subitizing range. There was no main effect of Color, *F*(1,23) = 3.49, *p* = 0.07. With these data, there was no significant interaction between the two factors, *F*(1,23) = 2.30, *p* = 0.14.

Accuracy rates were high when the number of stimuli fell within the subitizing range (*M*_Uniform Color_ = 0.91, *SD* = 0.11, *SE* = 0.02; *M* = 0.92_Disuniform Color_, *SD* = 0.12, *SE* = 0.02). Accuracy rates were lower when the number of stimuli fell outside of the subitizing range (*M* = 0.24_Uniform Color_, *SD* = 0.23, *SE* = 0.04; *M* = 0.21_Disuniform Color_, *SD* = 0.22, *SE* = 0.04). In a fully within-subjects ANOVA with Range (within versus outside) as one factor and Color (same versus different) as the other factor, there was a significant main effect on accuracy of Range, *F*(1,33) = 510.649, *p* < 0.0001, in which accuracy was higher when the objects were within the subitizing range than when the objects were outside the subitizing range. There was no main effect of Color, *F*(1,33) = 1.08, *p* = 0.31, and no significant interaction between the two factors, *F*(1,33) = 2.18, *p* = 0.15.

We examined the absolute difference between a subject’s count and the actual number of stimuli that were presented in the array. With this measure, it was revealed that subjects were more accurate when the number of stimuli fell within the subitizing range (*M* = 0.39_Uniform Color_, *SD* = 0.47, *SE* = 0.08; *M* = 0.35_Disuniform Color_, *SD* = 0.49, *SE* = 0.08) than when the number of stimuli fell outside this range (*M* = 5.72_Uniform Color_, *SD* = 2.01, *SE* = 0.34; *M* = 6.23_Disuniform Color_, *SD* = 1.78, *SE* = 0.31). With this measure, there was a significant main effect on accuracy of Range, *F*(1,33) = 424.30, *p* < 0.0001, in which accuracy was higher when the objects were within the subitizing range than when the objects were outside the subitizing range. There was a main effect of Color, *F*(1,33) = 6.33, *p* = 0.02, and a significant interaction between the two factors, *F*(1,33) = 8.88, *p* = 0.005.

#### Latency of Involuntary Counting

The mean latency of button-pressing was 1,348.65 ms (*SD* = 431.73, *SE* = 74.04) for the condition in which the objects were within the subitizing range (2–5 objects) and of uniform color. The mean latency was 1,423.78 ms (*SD* = 426.36, *SE* = 73.12) for the condition in which the objects were within the subitizing range (2–5 objects) and not of uniform color. When the number of objects were outside the subitizing range (i.e., 6–10 objects), latencies increased: 2,117.68 ms_Uniform Color_ (*SD* = 720.54, *SE* = 133.80), 2,253.96 ms_Disuniform Color_ (*SD* = 838.12, *SE* = 148.16).

In a fully within-subjects ANOVA with Range (within versus outside) as one factor and Color (same versus different) as the other factor, there was a main effect of Range, *F*(1,28) = 55.50, *p* < 0.0001, in which object arrays that were within the subitizing range (2–5 objects) were subitized at a faster rate than object arrays that were outside of the subitizing range (6–10 objects). There was no main effect of Color, *F*(1,28) = 1.31, *p* = 0.26, and no significant interaction between the two factors, *F*(1,28) = 0.023, *p* = 0.881. The null effect of the factor Color on latencies is consistent with the conclusions of Watson and Maylor (2006).

#### Perceived Immediacy of Involuntary Counting

When involuntary counting occurred, it was deemed to be immediate on a mean proportion of trials of 0.83 (*SD* = 0.16, *SE* = 0.03) for the condition in which the objects were within the subitizing range (2–5 objects) and of uniform color. The rate was 0.85 (*SD* = 0.14, *SE* = 0.02) for the condition in which the objects were within the subitizing range and not of uniform color. When the number of objects were outside the subitizing range (i.e., 6–10 objects), the rates of perceived immediacy decreased: 0.18_Uniform Color_ (*SD* = 0.19, *SE* = 0.03), 0.12_Disuniform Color_ (*SD* = 0.12, *SE* = 0.02).

In a fully within-subjects ANOVA with Range (within versus outside) as one factor and Color (same versus different) as the other factor, there was a significant main effect of Range, *F*(1,33) = 655.59, *p* < 0.0001, in which involuntary counting was more likely to be perceived as immediate in the subitizing condition than in the conditions in which the number of objects were outside the subitizing range. There was no main effect of Color *F*(1,33) = 2.95, *p* = 0.10, and an unexpected interaction between the two factors that will require replication and further investigation, *F*(1,33) = 6.69, *p* = 0.014. Again, this unexpected interactions effect does not qualify our primary conclusions in any substantive way.

#### Discussion

We provided a replication of the RIT in the context of the phenomenon of counting. The rate of involuntary counting was greater for object arrays within the subitizing range than for those outside of the subitizing range. Regardless of the task instruction (“*Do Not Count the Number of Objects*”), the RIT effect still arose on the majority of trials of the subitizing condition. This result suggests that subitizing is automatic and that the set to subitize is susceptible to external control. We did not find any differences in subitizing rates or latencies when the color of the arrays was manipulated experimentally. This corroborates what was found in a previous study (Watson and Maylor, 2006). We found that the effect was perceived more frequently to be “immediate” for object arrays within the subitizing range than for object arrays outside of the subitizing range.

In Experiment 1A, the experimenter somehow activated the set to count objects through the negative instructions (see Footnote 4). To the subject, the set to count was undesired and unselected, yet the set induced entry. Importantly, the set influenced conscious processing, and that which enters the conscious field, but it did not influence overt behavior. Consistent with this observation, it has been proposed that, during action selection, urges and other action-related thoughts can be construed as “action options” (Angell, 1907; Gibson, 1979; Morsella et al., 2016), which are the tokens of the selection process in voluntary action. In line with this view, Chomsky (1988) concluded that, unlike machines, humans are not only *compelled* to act in certain ways, but they can also be *inclined* to behave one way or another. For example, when performing Action *X*, one may also have conscious experiences about the inclination to perform Action *Y*, a state of affairs unlike anything that is instantiated in machines (as far as we can tell). Similarly, when buying a car, one may find oneself pondering about another car—the one that was roomier but, for some reason, was not selected for purchase (Filevich and Haggard, 2013).

**FIGURE 3:**
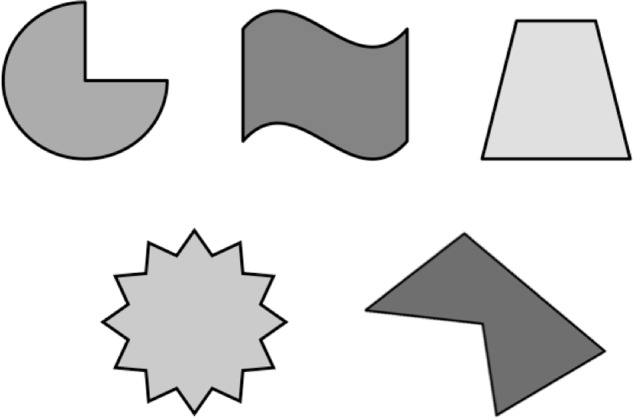
Stimuli used for Experiment 1B. Not drawn to scale.

In Experiment 1A, it was the experimenter who rendered the set to count to be an undesired action set. In everyday life, it is often one, the actor, who determines which sets are desirable or not. Would effects involving involuntary entry arise if it is the actor who selects which sets are undesirable and if the actor also selects, in the place of the undesired set, another, alternative set? We investigated this possibility in Experiment 1B, in which subjects were trained extensively on one set (e.g., to count objects) and then, when presented with stimuli (e.g., five nonsense objects; **Figure 3**), were given the choice to perform the trained set or a novel set. Will subjects experience both set-related imagery *and* set-unrelated imagery? If so, this would be consistent with theories proposing that the conscious contents represent several potential action plans (e.g., Morsella et al., 2016).

## Experiment 1B: Involuntary Entry From Unselected Sets

Subjects performed a block of 60 trials in which, at the beginning of each trial and before being presented with an array of stimuli (**Figure 3**), they could indicate whether they wanted to “color name” or “object count” during that trial. One hypothesis, *Conscious Content of Selected Set*, predicts that subjects would experience conscious thoughts associated only with the selected action set. According to this hypothesis, the mean proportion of set-unrelated thoughts should be zero or be statistically comparable to zero. The alternative hypothesis, *Conscious Content of Unselected Set*, predicts that, on a substantial proportion of the trials, subjects would experience conscious thoughts not only of the selected set but also of the unselected set. If the proportion of such thoughts is not comparable to zero, it remains an open question what the proportion would be. Do these involuntary thoughts occur on 10, 30, 50, or 80% of the trials?

When planning the experiment, we were concerned that there would be floor effects of set-unrelated imagery, for several reasons, including that the action plan of counting may be substantially weaker, at baseline, than the more frequently used plan of color naming. Hence, before the test phase of the experiment, subjects were exposed to an extensive set of trials in which they either color named or object named. This additional, between-subjects manipulation permitted additional contrasts and allowed us also to control to some extent subjects’ pre-test experiences with each of these action sets. For the sake of comparison, we took the opportunity to add a second block of trials in which the action set was determined, not by the subject, but externally (by computer program). This block was always presented as the second block, because we wanted subjects’ free selection of sets during the first, “Self-Select” block to be uninfluenced by any prior exposure to a set-selection process. For example, if subjects were presented in the first block to a regimen in which color-naming occurred on 50% of the trials, then this could influence set selection rates in the second, Self-Select block.

### Method

#### Subjects

Forty San Francisco State University students (*M*_Age_ = 24.38; *SD*_Age_ = 6.96; female = 29) participated for course credit.

#### Stimuli and Apparatus

We used the same hardware and software that was used in Experiment 1A. Latencies of the spoken responses were recorded via microphone (Model 33-3014; Radio Shack; Fort Worth, TX, United States), placed approximately 4 inches from subjects’ mouths. Four of the five nonsense stimuli were objects selected from *Shape* in Microsoft PowerPoint for Mac (2011), version 14.3.4 (**Figure 3**). The stimulus arrays of the objects varied on two dimensions: color [blue (RGB: 0, 0, 255), green (0, 255, 0), red (255, 0, 0), yellow (255, 255, 0), and purple (127, 0, 127)] and the number of objects presented on the screen (1, 2, 3, 4, and 5), yielding 125 unique arrays (i.e., 5 shapes × 5 colors × 5 numbers). The stimulus arrays were presented in the center of the screen with each object in the array having subtended visual angles of 5.96**°** × 5.96**°** (5 × 5 cm).

#### Procedures

All subjects first completed a training block with 375 trials. For training, subjects were randomly assigned to either the Color Naming condition (*n*= 20) or the Number Counting condition (*n*= 20). The stimulus arrays were the same in both conditions, but the manner in which the subjects responded to the arrays differed depending on the instructions. For Color Naming, subjects read the instructions, “*Please say the color of the object(s) into the microphone*.” For the Number Counting condition subjects read the instructions, “*Please say the number of object(s) into the microphone*.” Each training trial began with a ready prompt (a question mark) enabling subjects to decide when to begin the trial. Directly after subjects initiated the trial, a fixation cross (+) appeared for 500 ms in the middle of the screen, followed by the stimulus array, which remained on-screen until subjects spoke their response into the microphone (e.g., “green”), which initiated the next trial.

After training, subjects were informed that they would now be given the option to choose, on a trial-by-trial basis, either to name the color of the objects or count the number of objects (Self-Selected trials). Subjects were also informed that they would be responding to two mental imagery questions after viewing the object(s) on the screen. Examples of mental imagery and what it means to have visual and auditory imagery were displayed on the screen and read aloud by the experimenter to the subject (instructions were taken from Jantz et al., 2013). Regarding visual imagery, subjects read/heard, “*Take a moment to imagine what a tree looks like. Take a moment to imagine what a car looks like. You have just experienced an example of visual mental imagery*.” Regarding auditory imagery, subjects read/heard, “*Without saying it aloud, take a moment to imagine what the word ‘HOUSE’ sounds like. Take a moment to imagine what the word ‘FLOWER’ sounds like. You have just experienced an example of auditory mental imagery*.”

**FIGURE 4:**
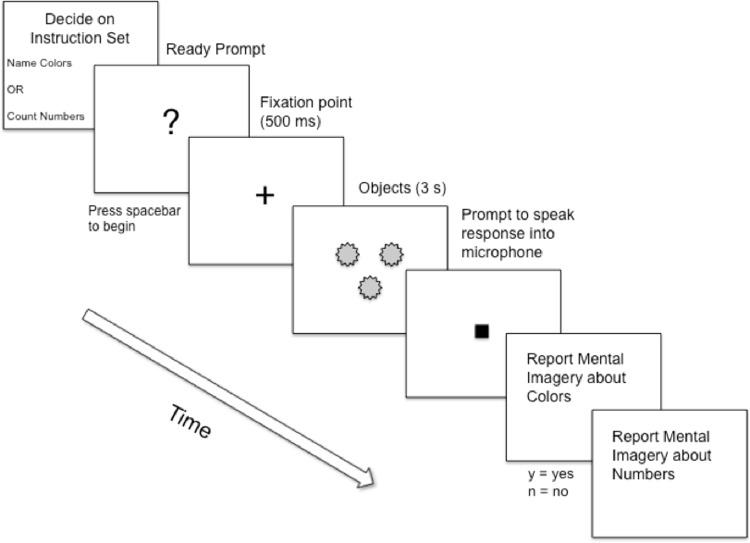
Schematic depiction of a typical trial (Experiment 1B). Not drawn to scale.

After the instructions, subjects first completed three practice trials and then 60 Self-Selected trials. Practice trials were exactly the same as the critical trials. During this time, the experimenter clarified any questions the subject had. At the beginning of each Self-Selected trial, subjects were asked, “*Please decide which instruction set you would like to follow: Name the color of the objects* or *Count the number of the objects.”* The subjects indicated their choice by pressing one of two keys on the keyboard. Each key had an overlay covering the surface of the key: The first overlay read *Count the Numbers*, and the second overlay read *Name the Colors*. The overlays covered keys “d” and “j” on the keyboard. The pairing of the two keys to the two overlays was fully counterbalanced across subjects. Thereafter, a ready prompt appeared. Once ready, subjects pressed the spacebar to begin the trial (**Figure 4**). Following initiation of the trial, a fixation point (+) appeared in the center of the screen (500 ms), followed by the presentation of the stimulus array (3 s). A cue then appeared indicating when subjects should utter their response into the microphone. During each experimental session, the experimenter observed the subject’s behavior and noted, on a trial-by-trial basis, whenever the subject emitted an incorrect response.

After a spoken response was made, subjects were asked two questions about their mental imagery: “*Did you experience any mental imagery related to colors?”* and “*Did you experience any mental imagery related to numbers?”* All subjects were told that these questions were in reference to the time during which the stimulus array was presented on the screen. The order of the presentation of these two questions was fully counterbalanced across subjects.

Following the block of Self-Selected trials, all subjects completed 60 Externally-Selected trials. These trials were exactly the same as the Self-Selected trials except that the instruction set was randomly selected for the subjects. The instruction set, indicating what kind of response to make, stated to either *“Count the number of object(s)”* or to *“Name the color of the object(s).”* This was followed by the mental imagery questions presented above.

Once subjects completed the experiment, they responded to a series of funneled debriefing questions (following the procedures of Bargh and Chartrand, 2000), which included general questions to assess whether (a) subjects were aware of the purpose of the study, (b) they had any strategies for completing the task, and (c) anything interfered with their performance on the task. Importantly, funneled debriefing also inquired whether subjects intended to follow the instruction set on each trial. Examination of the funneled debriefing data revealed that the subjects did not discern the hypothesis at hand and intended to follow the instruction set on each trial.

Based on previous research (Woodworth and Schlosberg, 1954; Morsella et al., 2009a,b), for the latency measure, response times faster than 200 ms or slower than 2,500 ms were excluded from analysis. The data from three subjects were excluded from analysis. The data from one subject were excluded because the subject was not able to finish the study in the allotted time and therefore did not complete the critical trials. The data from two other subjects were excluded because (a) it was obvious to the experimenter that these subjects did not follow directions and (b) there was over 20% data loss. For the remaining data, on 49 (1.0%) of the 4,800 trials, subjects did not answer the question about color imagery; on 56 (1.2%) of the 4,800 trials, subjects failed to provide an answer to the question about number imagery.

### Results

#### Selection of Set as a Dependent Measure

During the first block of trials, in which subjects could choose the action set (object counting versus color naming), subjects did not select one set significantly more often than the other. The raw proportions (*M*_Color Naming_ = 0.46, *SE* = 0.03; *M*_Object Counting_= 0.54, *SE* = 0.03) were comparable, *t*(39) = 1.22, *p* = 0.231, η_p_^2^ = 0.04. The same pattern of results was found with arcsine transformations of the proportions, *t*(39) = 0.96, *p* = 0.341 (arcsine transformations are often used to statistically normalize data that are in the form of proportions).

In a mixed design 2 × 2 ANOVA, with *proportion of set selection* as the dependent measure, Training (naming versus counting) as the between-subjects factor, and Set (color naming versus object counting) as the within-subjects factor, there was no main effect of Training, *F*(1,38) = 1.00, *p* = 0.324, no main effect of Set, *F*(1,38) = 1.46, *p* = 0.235, and no interaction between the two factors, *F*(1,38) = 0.46, *p* = 0.504. In an analysis with arcsine transformations of the proportions, the same pattern of results was obtained. There was no main effect of Training, *F*(1,38) = 1.42, *p* = 0.241, no main effect of Set, *F*(1,38) = 0.91, *p* = 0.346, and no interaction between the two factors, *F*(1,38) = 0.18, *p* = 0.677.

Analysis of the trial-by-trial data revealed that the average number of consecutive trials on which subjects would select the same set was ∼5 (e.g., *M* = 5.41 for the Color Set, *SD* = 10.62, *SE* = 1.68, Range = 0–60; and *M* = 6.32 for the Counting Set, *SD* = 12.79, *SE* = 2.02, Range 0–60). This mean number of consecutive trials was not influenced by training, the nature of the set (i.e., color naming versus object counting), or by the interaction of these factors, *F*s < 1, *p*s > 0.74. Only two subjects showed a strong bias in which the mean number of consecutive trials was more than 2 *SD*s above the mean of the 40 subjects.

#### Set-Unrelated Imagery

The most interesting finding was that, during the trials, set-unrelated imagery was experienced often by subjects (see this and other descriptive statistics in **Table 1**). During the block of trials in which subjects could freely select the set, the mean proportion of trials with set-unrelated imagery was 0.57 (*SE* = 0.04); during the second block of trials, in which the set was externally selected, the mean proportion was 0.54 (*SE* = 0.04). An ANOVA with Choice (Self-Selected versus Externally-Selected) as a within-subjects factor and Training (Color Naming versus Number Counting) as a between-subjects factor revealed that these proportions were uninfluenced by the factors Choice, *F*(1,38) = 0.78, *p* = 0.383, η_p_^2^ = 0.02, or Training, *F*(1,38) = 1.84, *p* = 0.183, η_p_^2^ = 0.05. There was an unexpected interaction between both factors, *F*(1,38) = 4.74, *p* = 0.04, η_p_^2^ = 0.11, in which Training yielded a difference only in the Externally-Selected condition. This unexpected difference, which requires further investigation, consisted of the training of Color Naming yielding less set-unrelated imagery than the training of Number Counting. Analysis of arcsine transformations of the data led to comparable results, with there being no main effect of Choice, *F*(1,38) = 0.44, *p* = 0.509, no main effect of Training, *F*(1,38) = 2.50, *p* = 0.122, and the aforementioned interaction between the two factors, *F*(1,38) = 5.15, *p* = 0.029.

**Table 1:** Mean Latencies (ms), proportions of imagery, and error rates as a function of block and training (Experiment 1B).

	All trials	Color training	Counting training
**Block 1: Self-selected set**
Set-related imagery	0.72 (0.05)	0.72 (0.06)	0.71 (0.07)
Set-unrelated imagery	0.57 (0.04)	0.56 (0.06)	0.58 (0.06)
Color imagery	0.62 (0.04)	0.62 (0.06)	0.62 (0.07)
Number imagery	0.66 (0.04)	0.66 (0.05)	0.67 (0.06)
Imagery of both sets	0.42 (0.05)	0.38 (0.06)	0.46 (0.07)
Error rates	0.01 (*SD* = 0.01)	0.004 (*SD* = 0.01)	0.01 (*SD* = 0.01)
Latencies	898.08 (33.50)	895.57 (53.84)	900.58 (41.33)
**Block 2: Externally-selected set**
Set-related imagery	0.73 (0.05)	0.79 (0.05)	0.67 (0.07)
Set-unrelated imagery	0.54 (0.04)	0.45 (0.05)	0.63 (0.06)
Color imagery	0.63 (0.04)	0.65 (0.04)	0.61 (0.07)
Number imagery	0.64 (0.04)	0.59 (0.04)	0.69 (0.07)
Imagery of both sets	0.38 (0.05)	0.31 (0.05)	0.46 (0.08)
Error rates	0.02 (*SD* = 0.03)	0.02 (*SD* = 0.02)	0.02 (*SD* = 0.03)
Latencies	843.29 (36.22)	829.35 (56.05)	857.24 (47.17)

*Note: Values in parentheses are SEs, unless otherwise indicated.*

#### Trials With Imagery From Both Sets

Importantly, the proportion of trials in which subjects explicitly reported the experience of both set-related and set-unrelated imagery was non-trivial and reliable. For the first block of trials, the proportion (*M* = 0.42, *SE* = 0.05) was significantly different from zero, *t*(39) = 8.98, *p* < 0.001. This was also the case for imagery during the second block of trials, in which the mean proportion was 0.38 (*SE* = 0.05), *t*(39) = 7.99, *p* < 0.001. Similar effects are found with the arcsine transformation of the proportion data, *t*s > 9.8, *p*s < 0.001. An omnibus ANOVA with Training as a between-subjects factor and Choice as a within-subjects factor revealed no main effect of Training, *F*(1,38) = 1.61, *p* = 0.213, no main effect of Choice, *F*(1,38) = 1.20, *p* = 0.281, and no interaction between the two factors, *F*(1,38) = 1.16, *p* = 0.289. The same pattern of results was found with arcsine transformations of the proportion data, *F*s < 1.82, *p*s > 0.185.

Regarding the experiences of color imagery versus number imagery, an ANOVA, along with the data presented in **Table 1**, revealed that, on average, the stimuli led to comparable rates of experienced color imagery and number imagery, *F*(1,38) = 0.43, *p* = 0.518. Across such imagery, there was no main effect of Choice, *F*(1,38) = 0.24, *p* = 0.629, and there were no significant interactions between experimental factors, *F*s < 4.05, *p*s > 0.05. The same pattern of null results was found with arcsine transformations of the proportion data, *F*s < 3.93, *p*s > 0.05.

#### Set-Related Imagery

An ANOVA with Training as a between-subjects factor and Choice as a within-subjects factor revealed that there was no main effect of Training, *F*(1,38) = 0.53, *p* = 0.47, no main effect of Choice, *F*(1,38) = 0.123, *p* = 0.728, and no interaction between the two factors on set-related imagery, *F*(1,38) = 2.76, *p* = 0.105. An analysis with arcsine transformations of the data revealed no main effect of Training, *F*(1,38) = 0.14, *p* = 0.708, no main effect of Choice, *F*(1,38) = 0.41, *p* = 0.526, and no interaction between the two factors, *F*(1,38) = 1.16, *p* = 0.289.

#### Set-Related Imagery Versus Set-Unrelated Imagery

As expected, for the Self-Select block (Block 1), the proportion of trials with set-related imagery (*M* = 0.72, *SE* = 0.05) was significantly higher than that of trials with set-unrelated imagery (*M* = 0.57, *SE* = 0.04), *t*(39) = 2.74, *p* = 0.009, η_p_^2^ = 0.16. The same effect was found with arcsine transformations of the proportion data, *t*(39) = 2.46, *p* = 0.018. Similar effects were also found with the proportion data from the second block, in which the set was externally selected, *t*(39) = 3.49, *p* = 0.001, and η_p_^2^ = 0.24. The same effect was found with the arcsine transformations of the data, *t*(39) = 3.41, *p* = 0.002. Consistent with these findings, an omnibus ANOVA with Training as a between-subjects factor and Set-Relation (Set-Related versus Set-Unrelated) and Choice (Self-Selected versus Externally-Selected) as within-subjects factors, there was a main effect of Set-Relation, *F*(1,38) = 15.69, *p* < 0.001, η_p_^2^ = 0.29, but no main effect of Training, *F*(1,38) = 0.075, *p* = 0.786, or of Choice, *F*(1,38) = 0.34, *p* = 0.566. There was an unexpected three-way interaction between the factors, *F*(1,38) = 5.27, *p* = 0.027, one in which effects of Training arose only in Block 2 (Externally-Selected condition), in which the training of Color Naming seems to have yielded less Set-Unrelated imagery (but not less Set-Related imagery) than the training of Number Counting. This unexpected interaction will require replication and further investigation.

#### Supplementary Data: Error Rates and Latencies

Mean error rates were very low (**Table 1**). There was no effect on error rates from the factor Training, *F*(1,38) = 0.13, *p* = 0.722, but there was an effect of the factor Choice, *F*(1,38) = 10.81, *p* = 0.002, which most likely reflects a carryover effect (e.g., fatigue) from Block 1. The same pattern of results was found with arcsine transformations of the error rate data. There was no interaction between the factors of Choice and Training, *F*(1,38) = 0.56, *p* = 0.458. Latencies were influenced by the factor Choice, *F*(1,38) = 19.12, *p* < 0.001, but not by Training, *F*(1,38) = 0.06, *p* = 0.814. It is parsimonious to propose that the decreased latencies for Block 2 reflect a carry over effect (e.g., a practice effect). There was no interaction between the two factors, *F*(1,38) = 0.83, *p* = 0.367.

#### Supplementary Analysis and Data Collection

Perhaps our pattern of results, in which set-unselected thoughts occurred for roughly 50% of the trials, arises only after subjects perform both sets over the course of several test trials. To examine this possibility, we examined subjects’ responses on the first trial of the first block. This analysis revealed that, across subjects, set-unrelated imagery occurred often during the first trial (*M* = 0.50, *SE* = 0.08), suggesting that our pattern of results could arise during the first trial, without subjects having experienced several trials. The mean proportion of first trials with imagery from both sets was 0.30 (*SE* = 0.07), and the mean proportion of trials with set-related imagery was 0.65 (*SE* = 0.08).

Supporting this interpretation, we conducted an additional, single-trial version of Experiment 1B with a different group of subjects [37 San Francisco State University students (*M*_Age_ = 22.27, *SD*_Age_ = 4.42; female = 25) who participated for course credit]. As in Experiment 1B, stimuli were presented on an Apple iMac computer monitor (50.8 cm) with a viewing distance of approximately 48 cm. The target stimulus was an array of three green squares displayed within a visual angle of 10.62° × 11.3**°** (8.89 × 9.52 cm) occupying the center of the screen. Before experiencing the first trial, subjects were instructed to either name the color or count the number of the objects that appeared on the screen. Subjects read the instructions, *“You will be shown an image on the screen. Please look at the image while it is on the screen.”* After subjects read the instructions, they moved to the next screen by pressing the spacebar. A fixation cross (+) appeared for 100 ms followed by the stimulus array for 3 s. Directly after the presentation of the stimulus array, subjects were asked three questions, (a) *While the image was on the screen, did you happen to think of the word “green”?* (b) *While the image was on the screen, did you happen to think of the word “three”?* (c) *While the image was on the screen, did you happen to think of the word “square”?* After each question, subjects reported their answer by either typing the letter *y* indicating “yes” or the letter *n* indicating “no.” The order of the questions was fully counterbalanced across subjects.

Across the 37 subjects, imagery of the three kinds occurred quite frequently: *M*_Color Imagery_ = 0.50 of the subjects (*SE* = 0.09), *M*_Number_ _Imagery_ = 0.69 (*SE* = 0.08), and *M*_Name Imagery_ = 0.72 (*SE* = 0.08).

Faced with these additional corroboratory data, one could argue that our pattern of results stemmed from subjects knowing *a priori* that they would be asked to perform only one of two sets and from subjects anticipating that they would be asked about whether they experienced any imagery associated with these sets. To examine this alternative hypothesis, we carried out an additional, single-trial version of Experiment 1B with a different group of subjects [36 San Francisco State University students (*M*_Age_ = 22.22, *SD*_Age_ = 8.58; female = 26) who participated for course credit]. In this experiment, before experiencing the first trial, subjects heard nothing about the two possible action sets. Subjects read the instructions, *“You will be shown an image on the screen. Please look at the image while it is on the screen.”* The target stimulus was an array of three green squares displayed within a 9.5 cm wide by 8.75 cm high area occupying the center of the screen. After subjects read the instructions they moved to the next screen by pressing the spacebar. A fixation point appeared for 500 ms followed by the stimulus array for 3 s. Directly after the presentation of the stimulus array, subjects were asked three questions, (a) *While the image was on the screen, did you happen to think of the word “green”?* (b) *While the image was on the screen, did you happen to think of the word “three”?* (c) *While the image was on the screen, did you happen to think of the word “square”?* After each question, subjects reported their answer by either typing the letter *y* indicating “yes” or the letter *n* indicating “no.” The order of the questions was fully counterbalanced across subjects.

Only 1 out of 36 subjects experienced no imagery of any kind, while many subjects experienced imagery of some kind: *M*_Color Imagery_ = 0.89 (*SE* = 0.05), *M*_Number_ _Imagery_ = 0.75 (*SE* = 0.07), and *M*_Name Imagery_ = 0.78 (*SE* = 0.07). Importantly, the proportion of subjects who experienced all three kinds of imagery was 0.58 (*SE* = 0.08).

In light of the findings from these two single-trial experiments, we entertained the possibility that, regarding the basic RIT effect (in which subjects cannot suppress the subvocalization of the names of visual objects presented to them), perhaps the effect will fail to arise if, before the presentation of the stimulus, subjects had activated the set, not to name objects, but rather to do something else (e.g., to name the colors in which stimulus words are presented). To investigate this possibility, we conducted another single-trial version of the RIT. In this experiment, which was based on the classic Stroop task (Stroop, 1935), another group of subjects (*n* = 45), was instructed to name the color in which letter strings were written in a series of 24 trials (importantly, these subjects had participated earlier that hour in an RIT study). There were three Stroop conditions, including Congruent (e.g., BLUE presented in blue), Incongruent (e.g., BLUE presented in red), and Neutral (e.g., XXXX presented in blue). Each condition consisted of eight trials. The 24 trials appeared in random order. Subjects were instructed to respond to each trial by speaking the answer into a microphone (Model 33-3013; Radio Shack; Fort Worth, TX, United States), placed approximately 2 inches from subjects’ mouths. RTs from spoken responses were recorded via microphone. To ensure that subjects were concentrating on the task, we had subjects respond after each trial to two questions using a keyboard: “*How strong was the urge to make a mistake*?” and *“How preoccupied are you with something other than the task at hand?”* Subjects responded using a 1 to 8 continuous scale, each with 1 indicating almost no urge or preoccupation, and 8 indicating extreme strong urge or preoccupation, respectively.

After the Stroop trials, subjects were presented with a prompt, “Name the Color of the Object” in which subjects indicated their response by speaking the answer into the microphone. Subjects were then presented with a line drawing of a colored table, based on the black-and-white line drawing of Snodgrass and Vanderwart (1980). Immediately following this trial, subjects were asked a question stating, “*During the last trial, when you were presented with the image of the object, did you happen to think of the object name (that is, “table”)*?” Subjects indicated their response by inputting *y* for “yes,” and *n* for “no” using the keyboard.

The results indicated that the RIT effect persisted for the majority of the subjects: a proportion of 0.80 (*SD* = 0.41, *SE* = 0.06) of the subjects still subvocalized the name of the object when they were presented with the object image even after they completed 24 trials of a task (the Stroop Task), which was concerned with the set of color naming, and even after being instructed to name the color of the object.

#### Discussion

Surprisingly, set-unrelated imagery occurred on roughly 50% of the trials of Experiment 1B, regardless of whether the set was self-selected or externally selected and regardless of whether subjects were trained to name colors or to count objects. The results are consistent with the *Conscious Content of Unselected Set* and inconsistent with the *Conscious Content of Selected Set*, in which conscious thoughts should be associated only with the selected action set. In addition, corroboratory evidence was found in an analysis of the first trial of the first block and in our supplementary data collection, which included “single-trial” versions of Experiment 1B. Complementing this evidence is the observation that, on over 37% of all the trials of Experiment 1B, subjects experienced conscious thoughts associated with both action sets (42% for Block 1 and 38% for Block 2). It is important to note that, in Experiment 1B, our trials provided sufficient time (3 s) for subjects to carry out two facile tasks—color naming and object counting. If the trials had been shorter, or if each of the tasks had been more challenging, set-unrelated imagery might have been substantially less frequent across trials.

Another limitation of Experiment 1B, including the single-trial variants of this experiment, is that we cannot corroborate that subjects’ self-reports about their subjective experience reflects what actually transpired during the trial. Some of the probe questions could be construed as “leading questions,” questions that increase the likelihood of artifacts from experimental demand. Future variants of the single-trial versions of Experiment 1B might be able to corroborate subjects’ self-reports by examining neural measures (e.g., the neural correlates of counting versus color-naming). In addition, future behavioral studies could employ questions that are more open-ended and that could not be construed as leading questions.

Consistent with theorizing (Morsella, 2005; Krisst et al., 2015), the data from Experiment 1B revealed that, when performing action *X*, people also experience conscious contents about action *Y*, even though the latter action was not selected for production. In our experiment, set-unrelated thoughts occurred on roughly half of the trials, regardless of whether the set was self-selected or selected externally. Importantly, such set-unrelated thoughts arose even though the experimenter never instructed subjects to have, or to not have, such thoughts.

### Experiment 2

Perhaps the involuntary counting that occurred in Experiment 1A arose only because the set to count objects was activated repeatedly by what was mentioned, regarding counting, after each trial. To provide some evidence against this alternative hypothesis, we conducted a single-trial version of Experiment 1A in which counting was not mentioned at all before the single trial. This variant of Experiment 1 would provide further evidence that subjects’ report of involuntary imagery in Experiment 1 stemmed from their knowledge of the actual number of objects presented in the stimulus array.

These data were collected from a different group of subjects (28 San Francisco State University students who participated for course credit). We used the same hardware and software that was used in Experiment 1A. The target stimulus was an array of nonsense stimuli displayed within a visual angle of 13.07° × 15.15**°** (11 × 13 cm) occupying the center of the screen. Instructions on the computer screen read, *“You will be shown an array of objects. Please keep your eyes focused on the center of the screen at all times.”* After subjects read the instructions, they moved to the next screen by pressing the return key. The stimulus arrays consisted of either three white nonsense objects (a sum below the subitizing range) or eight white nonsense objects (a sum above the subitizing range). Half of the subjects were presented with the former, and half of the subjects were presented with the latter.

Each object array appeared for 4 s. Directly after the presentation of the object arrays, subjects were asked two questions, (a) *If you counted the number of objects, how many objects did you count? If you did not happen to count the number of objects, you may indicate this by pressing ‘0.’* (b) *If you counted the number of objects, did you feel that the number came to mind immediately?* For this question, subjects reported their answer by either typing the letter *y* indicating “yes” or the letter *n* indicating “no.”

### Results

For the subjects (*n* = 14) presented with the array of three objects, which was a sum below the subitizing range, counting occurred for every single subject. Moreover, for each of these instances of counting, the counting was reported to be “immediate,” and was accurate (i.e., a count of three). For the subjects (*n* = 14) presented with the array of eight objects, which was above the subitizing range, counting occurred for a proportion of 0.36 of the subjects (*SD* = 0.50, *SE* = 0.13). The subjects who counted (*n* = 5), yielded the counts of *6, 6, 7, 7*, and *10*. Not one of the subjects presented with the array of eight objects reported that the counting was immediate. These results provide additional corroboration that the effect observed in Experiment 1A did not arise only because the set to count objects was activated repeatedly by what was mentioned, regarding counting, after each trial. However, one limitation of Experiment 2 is that, with the present data, one cannot corroborate subjects’ self-report of the counting imagery experienced during the trial. The first question following the trial could have increased the likelihood of experimental demand. Future variants of Experiment 2 might employ more open-ended questions or corroborate subjects’ self-reports by examining the neural measures associated with counting and subvocalization (e.g., activation in the superior temporal sulcus).

### General Discussion

The RIT reveals that, through the activation of sets, conscious thoughts can be triggered into existence by external stimuli in a manner that is nontrivial, principled, reliable, and systematic. Our experiments build on this past research by revealing how action sets can, when combined with certain forms of environmental stimulation, trigger the occurrence of high-level conscious thoughts, including those associated with counting, which is a sophisticated cognitive operation. In Experiment 1A, we provided a replication and extension of the RIT, one which illuminates further the nature of set-based entry. To our knowledge, this is one of the first demonstrations of the RIT involving the phenomenon of involuntary counting. Despite the task instruction (“Do Not Count the Number of Objects”), the involuntary counting still arose on a substantive proportion of the trials. Moreover, involuntary counting was more likely for object arrays within the subitizing range than for those outside of this range. Such an effect is unlikely to arise from experimental demand, for it would require for subjects to know how they should comport themselves in response to stimulus arrays that fall within the subitizing range and outside of this range.

It is parsimonious to conclude that the difference between the condition having 2–5 objects and the condition having more than five objects reflects a real occurrence of automatic, involuntary counting, a conclusion that is supported by the trial-by-trial immediacy measure provided by subjects. The immediacy measure revealed that involuntary counting was more likely to be perceived as immediate in the subitizing condition than in the conditions in which the number of objects were outside the subitizing range. The analyses did not reveal any differences in the latencies of involuntary counting when the color of the arrays was manipulated experimentally. Further research can be conducted to assess whether this will remain so with a larger sample size or with an experimental approach that is otherwise more sensitive.

One limitation of Experiment 1A is that it is challenging to create a variety of stimulus arrays that do not include recognizable patterns within them. Such patterns could influence perceptual grouping and the process of counting. For example, one of the stimulus arrays, composed of nine objects, was created in such a way that three objects were presented in rows of three. This might have led subjects to subitize the number *nine* by recognizing patterns of *three*.

Setting aside the limitations of the present project, it is important to emphasize that the RIT is the kind of paradigm that, because it builds incrementally on robust phenomena, has of recent been encouraged by leading researchers in the field (e.g., Nosek et al., 2012; Fiedler, 2017). The reliable, component processes of the RIT are of interest in disparate subfields of the study of mind and brain, including consciousness, attention, decision making, cognitive control, imagery, psychopathology, and action control.

It is also important to note that the kind of involuntary entry into consciousness found in the RIT arises also in tasks that lack any kind of negative instruction to not perform some kind of mental operation. For example, involuntary entry of contents into consciousness arises for ambiguous objects (e.g., Necker cube). In one experiment (Allen et al., 2016), subjects were instructed to hold in mind, for as long as possible, one way of perceiving an ambiguous object (e.g., Necker cube). Importantly, subjects were never told to *not* think about alternative ways in which the object could be perceived. Involuntary “perceptual reversals,” involving involuntary entry into consciousness of the rivalrous percept for a given object, occurred on around 80% of the trials, with roughly three such reversals per 30-sec trial.

Consistent with theorizing (Morsella, 2005; Morsella et al., 2016), the data from Experiment 1B revealed that, when performing action *X*, people also experience conscious contents about action *Y*, even though the latter action was not selected for production. In our experiment, set-unrelated thoughts occurred on roughly half of the trials, regardless of whether the set was self-selected or selected externally. Importantly, such set-unrelated thoughts arose even though the experimenter never instructed subjects to have, or to not have, such thoughts.

Interestingly, animal research may provide some additional evidence for the notion that representations of unselected actions are activated during action control. This has been demonstrated in the rat. Specifically, when the animal solves a T-maze by, say, taking the left path, there is activation of neural circuits associated with both this path and the incorrect path (e.g., to go right; Singer et al., 2013). Figuratively speaking, the neural evidence suggests that, though only one action was selected, both action options (i.e., the left and right paths) were entertained during deliberation. We find it interesting that our results are consistent with animal research revealing that, when an animal performs action *X*, representations of action *Y* may nonetheless be activated. In humans, such a multiplicity of conscious thoughts could arise in circumstances involving action options, such as the T-maze scenario mentioned above, or when one is purchasing a car or performing monotonous work.

In all of the present experiments, there was the involuntary entry of contents associated with action sets that were undesired or unselected by the subject (to compare the results across all the present studies, see **Table 2**). According to Ach (1910/2006), strong inclinations to execute a given action will arise when the action plan is challenged by some kind of obstacle or counter-force. This interesting hypothesis might explain the pattern of results displayed in **Table 2**, in which, for example, the imagery concerning counting was more prevalent across the trials of Experiment 1A (Subitizing Range condition), in which subjects were instructed to suppress counting, than across those of Experiment 1B, in which subjects were not instructed to suppress counting and the inclination to count was not challenged by so strong a counter-force. However, regarding this hypothesis, we must be conservative when comparing the results of Experiment 1A and Experiment 1B, for the two studies differ from each other regarding, not only the instructions, but many other respects (e.g., the nature of the stimuli).

**Table 2:** Imagery as a function of set, training, and mental operation: Counting, object naming, or color naming.

Involuntary counting	Mean proportion of trials
*Subitizing range*	
Uniform color (Exp. 1A)	0.90 (*SD =* 0.11, *SE* = 0.02, *Range* = 0.63–1.00)
Disuniform color (Exp. 1A)	0.91 (*SD =* 0.12, *SE* = 0.02, *Range* = 0.56–1.00)
Single-trial version (Exp. 2)	1.00
*Outside subitizing range*	
Uniform color (Exp. 1A)	0.24 (*SD =* 0.24, *SE =* 0.04, *Range =* 0–1.00)
Disuniform color (Exp. 1A)	0.19 (*SD =* 0.21, *SE =* 0.04, *Range =* 0–1.00)
Single-trial version (Exp. 2)	0.36 (*SD =* 0.50, *SE =* 0.13)
**Experiment 1B: Block 1: Self-selected set**
*All trials*	
Set-unrelated imagery	0.57 (*SD* = 0.26, *SE* = 0.04, *Range* = 0.09–1.00)
*Color training*	
Set-unrelated imagery	0.56 (*SD* = 0.26, *SE* = 0.06, *Range* = 0.09–1.00)
*Counting training*	
Set-unrelated imagery	0.58 (*SD* = 0.27, *SE* = 0.06, *Range* = 0.12–1.00)
**Experiment 1B: Block 2: Externally-selected set**
*All trials*	
Set-unrelated imagery	0.54 (*SD* = 0.27, *SE* = 0.04, *Range* = 0–1.00)
*Color training*	
Set-unrelated imagery	0.45 (*SD* = 0.24, *SE* = 0.05, *Range* = 0–0.89)
*Counting training*	
Set-unrelated imagery	0.63 (*SD* = 0.27, *SE* = 0.06, *Range* = 0.12–1.00)
**Incidental imagery in the single-trial version of Exp. 1B**
Color imagery	0.89 (*SD* = 32, *SE =* 0.5)
Number Imagery	0.75 (*SD =* 44, *SE =* 0.7)
Name imagery	0.78 (*SD =* 42, *SE =* 0.7)
**Incidental name imagery following color naming in Stroop Task single-Trial)**
Name imagery	0.80 (*SD =* 0.41, *SE =* 0.06)

The findings presented in **Table 2** are consistent with theories proposing that, during action control, conscious contents function as “action options” for voluntary behavior (Morsella, 2005; Morsella et al., 2016). As evident in our experiments, these contents are often generated in an “encapsulated” manner. Theorists have posited that conscious contents arise involuntarily because of the encapsulated nature of the generation of most conscious contents (Fodor, 1983; Morsella et al., 2016). Perceptual processes giving rise to illusions are often said to be encapsulated, because knowledge of the true nature of the perceptual stimuli (e.g., that the two lines of the Müller-Lyer illusion are equal in length) cannot ‘turn off’ the illusion. This encapsulation is evident in perception and also in the generation of action-related urges. In certain stimulus environments, these urges (e.g., to inhale while holding one’s breath while underwater) are triggered in a predictable and insuppressible manner (Morsella, 2005). The urges cannot be modulated or turned off voluntarily, even when doing so would be adaptive (Öhman and Mineka, 2001; Morsella, 2005). The action-related urges are externally-triggered and encapsulated from volitional processes. As noted by Bargh and Morsella (2008), these action-related inclinations can be *behaviorally suppressed*, but they often cannot be *mentally suppressed*.

More generally, the present RIT effects reveal that the generation of conscious contents, one of the greatest mysteries in science (Crick and Koch, 2003; Dehaene, 2014; Koch et al., 2016), can be studied experimentally by the systematic activation of action sets. The effects on consciousness from these sets are reliable, robust, and predictable. The unpredictability of entry encountered in everyday life may reflect, not so much the working of an un-mechanistic system and indeterminate system, but rather the vagaries of both the transient activations of action sets, whether they are activated by external stimuli or by memorial processes, and concurrent stimulus conditions. Under the correct conditions, entry can be highly predictable and externally constrained.

## Author Contributions

SB conducted Experiment 1A, Experiment 2, and the some of the supplemental data collection. SB also assisted with all of the other projects. CM conducted Experiment 1B and some of the supplemental data collection. CM also assisted with the other projects. HC conducted some of the experiments for the supplemental data collection and assisted with the other projects. EM assisted with the design of the studies and the data analyses. All authors contributed to the analyses and to the writing of the manuscript.

## Conflict of Interest Statement

The authors declare that the research was conducted in the absence of any commercial or financial relationships that could be construed as a potential conflict of interest.

## Footnotes

1 A “conscious content” is any thing that one is aware of (Merker, 2007); for example, it might be a color, an urge, or a spontaneous autobiographical memory. The “conscious field” is all that one is aware of at one moment in time, which is the combination of all activated conscious contents (Köhler, 1947; Searle, 2000; Freeman, 2004).

2 This RIT effect requires the process of object naming, a sophisticated, multi-stage process in which only one of tens of thousands of phonological representations is selected for production in response to a stimulus (e.g., CAT yields /k/, /æ/, and /t/; Levelt, 1989). After the presentation of the stimulus, the RIT effect arises after a few moments [*M* = 1,451.27 ms (*SD* = 611.42) in Allen et al., 2013b; *M* = 2,323.91 ms (*SD* = 1,183.01) in Cho et al., 2014; *M* = 1,745.97 ms (*SD* = 620.86) in Merrick et al., 2015].

3 One of the many differences between the involuntary subvocalization that constitutes the RIT effect and the kinds of effects that have been obtained in most experiments concerning ironic processing is that, in the latter, subjects are presented with a verbal description (e.g., verbal instructions such as “*Do not think of white bears*”), and then the subjects experience involuntary perceptuo-semantic imagery. In the RIT, however, the stimuli are visual, and it is the involuntary imagery that is phonological in nature. One could state that the RIT involves the opposite direction of activation of that found in the classic studies on ironic processing.

4 It is unlikely that subjects participating in a basic version of the RIT would experience the phonological representations of the names of the objects that are perceived visually without first having activated somehow the set to name objects. The activation of the set is somehow initiated by the instruction to *not* think of the name of the object. This is a *negative instruction* which could have peculiar effects. Because of this possibility, in Allen et al. (2013b), there was an *Incidental Naming* condition in which subjects were not provided with the ‘do not think’ instruction that leads to ironic effects (Wegner, 1994). Instead, the condition involved no explicit instructions regarding naming or not naming. For this condition, involuntary subvocalization of the object names still arose on 99% of the trials (range = 80–100%). The effect was comparable even on the very first trial [31 (97%) out of 32 first trials]. The Incidental Naming condition served to evaluate subjects’ spontaneous subvocalization rates in response to the stimuli when having no obvious action set toward the stimuli. Of course, simply mentioning to subjects the possibility of naming will increase the likelihood of subvocalization, which is a limitation of this condition (see also, in General Discussion, treatment of Allen et al., 2016, which was another RIT that did not have negative instructions).

## Appendix

**Table:**

Object Range	Visual Angle
2	6.56° × 9.53° (5.5 × 8 cm)
3	9.53° × 9.53° (8 × 8 cm)
4	8.34° × 10.71° (7 × 9 cm)
5	10.71° × 14.25° (9 × 12 cm)
6	11.89° × 14.84° (10 × 12.5 cm)
7	11.89° × 14.25° (10 × 12 cm)
8	13.07° × 15.15° (11 × 13 cm)
9	14.25° × 15.15° (12 × 13 cm)
10	11.89° × 16.59° (10 × 14 cm)
